# Gastrointestinal carriage of carbapenemase-producing enterobacterales among inpatient and outpatient children in Kenya

**DOI:** 10.1038/s41598-024-78059-1

**Published:** 2024-12-28

**Authors:** Susan Githii, John M. Maingi, Teresia Nyaga, Cecilia Ndungu, Kelvin Wangira Nyongesa, Abednego Moki Musyoki

**Affiliations:** 1 National Public Health Laboratory, Upperhill, Kenyatta National Hospital Grounds, 20750-00202 Nairobi, Kenya; 2 Department of Biochemistry, Microbiology and Biotechnology, School of Pure and Applied Sciences, Kenyatta University, 43844-00100 Nairobi, Kenya; 3 Department of Medical Laboratory, Murang’a County Referral Hospital, 69-10200 Murang’a, Kenya; 4 Department of Medical Research, Kenyatta National Hospital, P.O Box 20723-00202, Nairobi, Kenya; 5 Department of Medical Laboratory Science, School of Health Sciences, Kenyatta University, 43844-00100 Nairobi, Kenya

**Keywords:** Gastrointestinal carriage, Carbapenem-resistant Enterobacterales, Carbapenemase-producing enterobacterales, Multidrug-resistant, Risk factors, Medical research, Risk factors

## Abstract

Gastrointestinal carriage of antimicrobial-resistant bacteria, especially carbapenemase-producing Enterobacterales (CPE), presents a critical public health threat globally. However, in many resource-constrained countries, epidemiological data on CPE is limited. Here, we assessed gastrointestinal carriage and associated factors of CPE among inpatient and outpatient children (≤ 5 years). This hospital-based cross-sectional study was conducted at Thika Level 5 Hospital in Kenya from February to June 2023. In total, 540 participants were recruited from outpatient (270) and inpatient (270) children, excluding those admitted for < 48 h and outpatients with ≤ 3 months hospitalization history. Demographic data were collected using a questionnaire, and stool or swab samples were cultured following standard microbiology methods. Automated platforms were used for isolates identification and antimicrobial susceptibility testing. Gastrointestinal carriage rate of CPE was 9.6%, 95% confidence interval (CI): 6.39–13.79% (26/270) among the inpatients and 5.9%, 95% CI: 3.42–9.45% (16/270) among the outpatients. The carbapenemase-producing Enterobacterales were predominated by multidrug-resistant *Escherichia coli* from inpatients (22/22, 100%) and outpatients (8/10, 80%). The colonization rate was higher among inpatients who were presenting with chills (aOR = 10.57, *p* = 0.008). We report a high gastrointestinal carriage of CPE among children (≤ 5 years). Strict adherence to colonization screening, and antimicrobial stewardship policies are critical to control CPE dissemination in the current study area and beyond.

**Supplementary Information:**

The online version contains supplementary material available at 10.1038/s41598-024-78059-1.

## Introduction

Antimicrobial resistance (AMR) remains a formidable public health challenge globally. In 2019, there were an estimated 4.95 million deaths associated with bacterial AMR, including 1.27 million attributable deaths, with the burden disproportionately higher in Sub-Saharan Africa^1^. Worsening of the situation is expected by 2050, with a projected yearly burden of 10 million deaths and 100 billion US dollars in economic loss^2^. Antimicrobial-resistant bacteria, particularly carbapenem-resistant strains, are frequently associated with high morbidity, increased length of hospital stay, and mortality^3^. Since their introduction in the early 1980s, carbapenems emerged as the preferred treatment for bacterial infections, especially those caused by multidrug-resistant (MDR) Gram-negative bacteria (GNB), due to their high effectiveness and minimal toxicity^4^. In recent years, however, the global surge in resistance, attributed to its overuse and misuse, has negatively affected the clinical relevance of carbapenems^5^. To date, carbapenem-resistant bacteria, including Enterobacterales, *Pseudomonas aeruginosa*, and *Acinetobacter baumannii*, tops the list of World Health Organization (WHO) global priority pathogens for the development of newer antimicrobial agents^6^.

Carbapenem resistance occurs through decreased membrane permeability, overexpression of efflux pumps, drug target modification, and production of carbapenemases. Carbapenemases are chromosomally or plasmid-encoded enzymes that hydrolyze the carbapenems and are the chief resistance mechanism in this antibiotic class^7^. The global clinically significant carbapenemases, including *Klebsiella pneumoniae* Carbapenemase (KPC), New Delhi metallo-β-lactamase-type 1 (NDM-1), Verona Integron Encoded Metallo-β-Lactamase (VIM), and Imipenemase Metallo-β-Lactamase (IMP)^8^ are generally located within different integrons associated with mobile plasmids or transposons, facilitating the transfer of resistance genes between bacteria. Oxacillinase (OXA)-48 and its related variants are also globally relevant because of their β-lactamase inhibitor resistance^8^.

Carbapenem-resistant (CR) bacteria are frequently MDR, which substantially limits therapeutic options and are associated with high mortality rates (33–50%)^9–12^. The triple combination of polymyxin, carbapenem, and rifampin or tigecycline, as an option, is faced with efficacy uncertainty due to emerging resistance. The newer antibiotics, including ceftazidime-avibactam, ceftolozane-tazobactam, meropenem-vaborbactam, imipenem-cilastatin-sulbactam, plazomicin, eravacycline, and cefiderocol, are expensive, have limited high-quality clinical data, and are faced with delays in susceptibility testing methods approval and the complexity of antibacterial spectra^13^.

Despite the adoption of the Global Action Plan on AMR by the World Health Assembly in May 2015, carbapenem resistance in Africa is seemingly increasing, with reported rates ranging from less than 1–60% among GNB^14–16^. The gastrointestinal tract can serve as a hidden reservoir for carbapenem-resistant Enterobacterales (CRE), such as *Klebsiella*,* Escherichia*,* Aeromonas*,* Serratia*, *Citrobacter,* *Proteus*, *Morganella*,* Providencia*, and *Enterobacter.* Asymptomatic CRE colonization may precede infection among susceptible persons, including those in intensive care units (ICU), artificially ventilated, catheterized, and with weakened immunity^17^, and has been identified as a significant risk factor for the development of CRE infections^18^. Colonization can also increase the risk of the resistance traits disseminating to other bacteria, escalating AMR.

The predictors of CRE colonization are exposure to antibiotics such as carbapenems, cephalosporins, fluoroquinolones and vancomycin, presence of an invasive device, number of hospital admissions, admission from another facility, and admission from or discharge to a long-term care facility^17^. However, in many Sub-Saharan African countries, especially in Kenya^13^, with inadequate potable water, poor sanitation, poor adherence to antimicrobial stewardship policies, and the majority of healthcare facilities lacking microbiology laboratories capable of reporting AMR, there is limited data on gastrointestinal CRE carriage to inform infection prevention and control interventions. Here, we aimed to determine the gastrointestinal carriage of CPE and risk factors among children under five years in inpatient and outpatient departments in a Kenyan hospital.

## Materials and methods

### Study setting, design, and population

The study was conducted at Thika Level 5 Hospital, located in Thika town, approximately 50 km from Nairobi City, Kenya, and serving as a referral facility for Kiambu County. The facility has a 467-bed capacity, serves an average of 800 to 1000 outpatients daily, and receives patients from neighbouring counties, including Nairobi City, Muranga, Kirinyaga, and Machakos. It was a cross-sectional study among children (≤ 5 years) admitted to the hospital’s pediatric ward and those seeking healthcare services in the outpatient department, whereby consecutive and systematic random sampling techniques, respectively, were employed. We included participants with or without gastrointestinal illnesses, excluding outpatients with a hospitalization history within three months and inpatients admitted for less than 48 h. The research was carried out following the Declaration of Helsinki, obtaining informed consent from each participating child’s parent or guardian, ensuring the participants’ well-being, and promptly communicating all critical results with physicians. The participant’s data and samples were assigned unique codes to ensure confidentiality.

### Samples collection

A structured questionnaire, administered through face-to-face interviews, was used to collect the participants’ demographic and clinical characteristics data for putative CRE carriage risk factors analysis. Stool samples, collected in clean and dry wide-necked containers, and rectal swabs collected in Cary-Blair transportation medium (Hopebio, Qingdao, China) from children unable to provide stool were transported in a cool box to the Thika Level 5 Hospital microbiology laboratory for processing within two hour-holding time.

### Phenotypic screening of carbapenemase-producing Enterobacterales

Initially, we screened stool/swab samples for carbapenem-resistant Enterobacterales (CRE) using MacConkey agar supplemented with 1 µg/ml of meropenem and incubated at 37 °C for 18–24 h^19^. Growth of colonies on the antibiotic-supplemented MacConkey agar was interpreted as positive-CRE carriage, whereas the absence of growth was considered CRE carriage-negative. *E. coli* ATCC 25,922 and *Klebsiella pneumoniae* ATCC 700,603 were the positive and negative control organisms, respectively.

The CRE positive isolates were confirmed for carbapenemase production using the modified carbapenem inactivation method (mCIM) following the CLSI (2023) guidelines^20^. The test isolate was transferred from an overnight culture using a sterile wire loop into a tube containing 2 ml Tryptic Soy Broth (TSB). The suspension was vortexed for 10 min, a 10 µg meropenem disk immersed, and incubated for 4 h at 35 °C. *E. coli* ATCC 25,922 suspensions, equivalent to 0.5 McFarland standard, was prepared using normal saline, inoculated on Muller Hinton Agar (MHA) plates, and air-dried for 10 min. Subsequently, we removed the meropenem disc from the test isolate-TSB culture using a sterile 10 µL loop to the MHA and incubated the plate for 18 to 24 h at 35 °C.

Isolates with 6–15 mm inhibition zone diameter were interpreted as carbapenemase (producers) positive, considered to have produced carbapenemase enzyme that hydrolyzed the meropenem antibiotic, resulting in no inhibition or limited growth inhibition of the meropenem-susceptible *E. coli* ATCC 25,922. A ≥ 19 mm inhibition zone diameter was interpreted as carbapenemase negative, indicating that the test isolate did not produce the carbapenemase to hydrolyze the meropenem disk, resulting in growth inhibition of the meropenem-susceptible *E. coli*. Isolates with 16–18 mm and ≥ 19 mm inhibition zone diameters and pinpoint colonies were considered indeterminate, and their mCIM test was repeated^20^. We used *E. coli* ATCC 25,922 (non-carbapenemase producer) and *Klebsiella pneumoniae* ATCC 700,603 (carbapenemase-producer) as the quality control organisms.

### Isolates identification and antibiotic susceptibility testing

The isolates that turned positive for carbapenemase production were identified using matrix-assisted laser desorption ionization-time of flight mass spectrometry ((MALDI-TOF MS) (Bruker Daltonik GmbH, Bremen, Germany). VITEK 2 (bioMK2 bi S.A., NS. A, Nu, Germany) was used for antimicrobial susceptibility testing. The choice and interpretation of antimicrobial tested, including cefepime, ceftriaxone, cefuroxime, ceftazidime, cefotaxime, meropenem, ciprofloxacin, gentamicin, amikacin, colistin, trimethoprim-sulfamethoxazole, ampicillin, and piperacillin-tazobactam, were based on CLSI (2023) guidelines. *Pseudomonas aeruginosa* (ATCC 27853) and *Escherichia coli* (25922) served as the quality control organisms^20^.

### Colistin susceptibility testing

This study used the broth microdilution (BMD) method for colistin susceptibility testing, as recommended by the CLSI 2023. Four tubes containing calcium magnesium Muller Hinton Broth were labelled 1,2, 4 µg/ml and 0 µg/ml (control), corresponding to colistin discs added, and the tubes were gently vortexed for 30 min to allow the colistin to elute from the disc. We prepared a 0.5 MacFarland standard equivalent suspension of each test organism, and sequentially 50 µl added to tubes 0, 1, 2 and 4 µg/ml, plated the original inoculum on a blood agar (BA) plate using a ten (10) µl loop for purity check, and incubated the plates and tubes at 35 °C for 16–20 hours. For the test to be valid, colonies on the BA plate were to be pure with the control tube (0 µg/ml) showing turbidity. The minimum inhibitory concentration (MIC) of ≤ 2 µg/ml was considered intermediate and ≥ 4 µg/ml resistant, as per the CLSI, 2023, which does not provide colistin “susceptible” interpretation due to variability in testing methods and clinical outcomes. *E. coli* ATCC 25,922 and *Proteus mirabilis* were used as control organisms.

### Statistical analysis

We analyzed the data obtained using Stata version 13 (STATA Corporation, College Station, TX, USA). The data was analyzed for normality using Shapiro-Wilk test. The frequencies and percentages were used to present the categorical data, while the means and medians were used for the continuous data, and these were displayed in tables and figures. The study used bivariable logistic regression to determine the association between the CPE gastrointestinal carriage and the participant’s demographic and clinical characteristics. Variables having a p-value ≤ 0.2 were subjected to further analysis by multivariable logistic regression to assess the independence of the associations, with p-value ≤ 0.05 and a corresponding 95% confidence interval (CI) considered statistically significant. We defined multidrug resistance as resistance to at least one antibiotic in at least three antimicrobial classes^21^ and multiple antibiotic resistance indices (MARI) as a ratio of the number of antibiotics to which a bacterial isolate was resistant and the total number of antibiotics tested^22^.

## Results

### Demographic and clinical characteristics of study participants

This study sampled a total of 540 children (≤ 5 years), equally drawn from outpatients and inpatients. The majority of the participants were males (outpatients: 53.3%, 144/270; inpatients: 50.4%, 136/270), aged ≤ 24 months (outpatients: 73.3%, 198/270; inpatients: 76.7%, 207/270) and had antibiotics exposure within three months of the sample collection (inpatients: 54.1%, 146/270; outpatients: 71.1%, 192/270), Table 1.

**Table 1:** Demographic and clinical characteristics of study participants.

Variable	Category	Proportion, *n* (%)	
		Inpatients	Outpatients
Age			
	≤ 24 months	207 (76.7)	198 (73.3)
	> 24 months	63 (23.3)	72 (26.7)
Gender			
	Male	136 (50.4)	144 (53.3)
	Female	134 (49.6)	126 (46.7)
Clinical presentation			
	Vomiting	49 (18.1)	99 (36.7)
	Chills	3 (1.1)	13 (4.8)
	Diarrhoea	42 (15.6)	97 (35.9)
	Headache	3 (1.1)	2 (0.7)
	Respiratory illness	76 (28.1)	144 (53.3)
	Fever	68 (25.4)	136 (50.6)
Antibiotics use in the past 3 months			
	Yes	146 (54.1)	192 (71.1)
	No	124 (45.9)	78 (28.9)
Source of antibiotics			
	Chemist	37 (25.3)	35 (13.0)
	Healthcare facility	109 (74.7)	157 (58.1)
Used antibiotics prescribed by the clinician			
	Yes	83 (30.7)	87 (32.2)
	No	187 (69.3)	183 (67.8)
Completed the last antibiotic dose treatment			
	Yes	196 (72.6)	108 (39.6)
	No	74 (27.4)	162 (60.4)
Source of domestic water			
	Municipal water	254 (94.1)	241 (89.3)
	Borehole water	16 (5.9)	29 (10.7)

### CPE gastrointestinal carriage

**Fig. 1:**
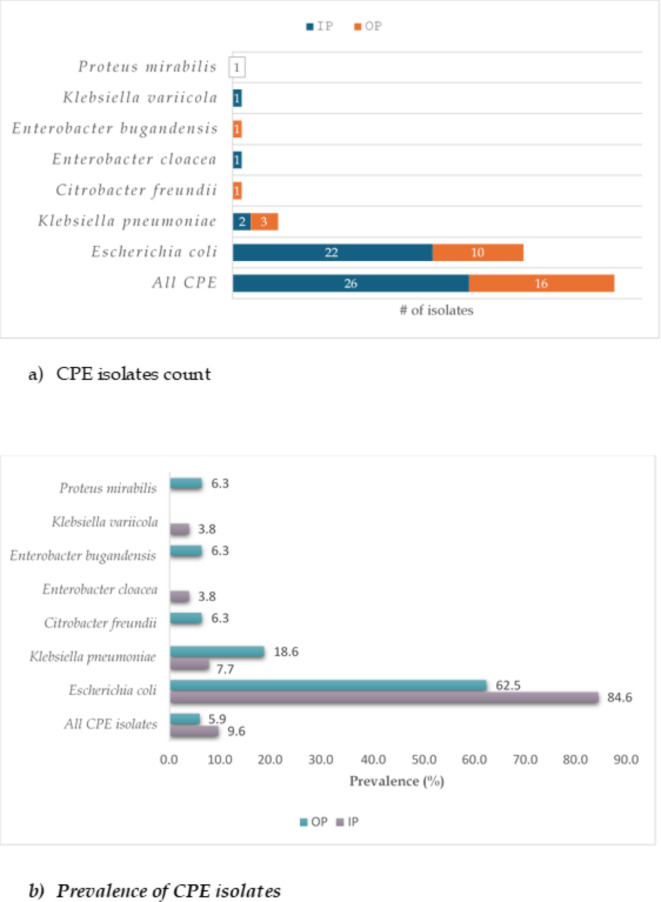
Gastrointestinal carriage of CPE among the study participants. * IP* inpatients,* OP* outpatients, # number, % percent,* CPE* carbapenem-resistant Enterobacterales.

Gastrointestinal carriage rate of CPE was 9.6%, 95% confidence interval (CI): 6.39–13.79% (26/270) among the inpatients and 5.9%, 95% CI: 3.42–9.45% (16/270) among the outpatients, Fig. 1b. All the carbapenem-resistant Enterobacterales (CRE) isolates were carbapenemase producers. The spectrum of the carbapenemase-producing Enterobacterales (CPE) included five bacterial species recovered from outpatients and four from inpatients and was predominated by carbapenemase-producing *Escherichia coli* (CPEC) (inpatients: 22/26, 84.6%; outpatients: 10/16, 62.5%)), Fig. 1a. There was a co-carriage of *E. coli* and *Klebsiella pneumoniae* among inpatients (1/270, 0.4%). CPE, including *Enterobacter cloacae* and *Klebsiella variicola*, were not isolated from outpatients, whereas *Citrobacter freundii*,* Enterobacter bugandensis* and *Proteus mirabilis* were absent among the hospitalized children, Fig. 1a.

### CPE antimicrobial susceptibility profiles

Here, we presented the antimicrobial susceptibility profile of carbapenemase-producing *E. coli* (CPEC) isolates, as there were only a few proportions of the other CPE isolates whose analysis could have yielded unreliable conclusions. Resistance to third-generation cephalosporins (3GCs) was 100% in both inpatient and outpatient children, Table 2. Aminoglycosides resistance was highest in CPEC isolates from inpatients (32%) compared with those from outpatients (10%), whereas the non-susceptibility to monobactam (aztreonam) was observed at 100% among outpatients and inpatients. Generally, the lowest antimicrobial resistance was in aminoglycosides (10–32%), and all CPEC isolates were susceptible to colistin, Table 2.

**Table 2:** Antimicrobial susceptibility profiles of carbapenemase-producing *E. coli*.

Antibiotics class	Antibiotics	Phenotype	Outpatients n = 10	Inpatients n = 22
Penicillin	AMP	R	100%	100%
	TZP	R	40%	45%
2GC	CXM	R	100%	100%
3GC	CTX	R	100%	100%
	CAZ	R	100%	100%
	CRO	R	100%	100%
4GC	FEP	R	60%	91%
AMINO	AMK	R	10%	32%
	GEN	R	10%	32%
Quinolones	CIP	R	50%	50%
Sulfonamides	SXT	R	100%	95%
Carbapenem	MEM	R	100%	100%
Polymyxins	CST	R	0%	0%
Monobactam	ATM	R	100%	100%

*AMP* ampicillin,* TZP* piperacillin/tazobactam,* CXM* cefuroxime,* CTX* cefotaxime,* CAZ* ceftazidime,* CRO* ceftriaxone,* FEP* cefepime,* AMK* amikacin,* GEN* gentamicin,* CIP* ciprofloxacin,* SXT* trimethoprim/sulfamethoxazole,* MEM* meropenem,* CST* colistin,* ATM* aztreonam,* S* susceptible,* R* resistant,* 2GC* second-generation cephalosporin,* 3GC* third-generation cephalosporin,* 4GC* fourth-generation cephalosporin.

### MDR carbapenemase-producing *E. coli* isolates

All CPEC isolates from inpatient (22/22, 100%) and 80% of those recovered from outpatient (8/10) were multidrug-resistant but remained susceptible to colistin, Table 3. Multiple antibiotic resistance indices (MARI) were high, ranging from 0.23 to 0.46.

**Table 3:** MDR carbapenemase-producing *E. coli* isolates.

Multidrug resistance phenotype	MDR isolates		#	MARI
	IP, *n* (%)	OP, *n* (%)		
AMP-TZP/CXM-CTX-CAZ-CRO-FEP/MEM/SXT	1 (4.5)	2 (20.0)	4	0.31
AMP-TZP/CXM-CTX-CAZ-CRO-FEP/CIP/SXT	1 (4.5)	1 (10.0)	4	0.31
AMP/CXM-CTX-CAZ-CRO-FEP/MEM/CIP/SXT	1 (4.5)	1 (10.0)	5	0.38
AMP/CXM-CTX-CAZ-CRO-FEP/MEM/AMK-GEN/CIP/SXT	1 (4.5)	1 (10.0)	6	0.46
AMP-TZP/CXM-CTX-CAZ-CRO-FEP/MEM/AMK-GEN/CIP/SXT	4 (18.2)	0 (0.0)	6	0.46
AMP/CXM-CTX-CAZ-CRO-FEP/MEM/SXT	3 (13.6)	0 (0.0)	4	0.31
AMP/CXM-CTX/SXT	3 (13.6)	0 (0.0)	3	0.23
AMP-TZP/CXM-CTX-CAZ-CRO-FEP/MEM/CIP/SXT	2 (9.1)	0 (0.0)	5	0.38
AMP/CXM-CTX-CAZ-CRO-FEP/SXT	2 (9.1)	0 (0.0)	3	0.23
AMP/CXM-CTX-CAZ-CRO-FEP/MEM/GEN/SXT	1 (4.5)	0 (0.0)	5	0.38
AMP/CXM-CTX/MEM/SXT	1 (4.5)	0 (0.0)	4	0.31
AMP/CXM-CTX-CAZ-CRO-FEP/MEM/AMK/CIP/SXT	1 (4.5)	0 (0.0)	5	0.38
AMP-TZP/CXM-CTX-CAZ-CRO-FEP/MEM/AMK-GEN/SXT	1 (4.5)	0 (0.0)	5	0.38
AMP/CXM-CTX-CAZ-CRO/CIP/SXT	0 (0.0)	2 (20.0)	4	0.31
AMP/CXM-CTXCAZ-CRO-FEP/SXT	0 (0.0)	1(10.0)	3	0.23
MDR, n (%)	22 (100.0)	8 (80.0)		

*AMP* ampicillin,* TZP* piperacillin/tazobactam,* CRX* cefuroxime,* CXM* cefuroxime,* CTX* cefotaxime,* CAZ* ceftazidime,* CRO* ceftriaxone,* FEP* cefepime,* AMK* amikacin,* GEN* gentamicin,* CIP* ciprofloxacin,* SXT* trimethoprim/sulfamethoxazole,* CST* colistin, # Number of antimicrobial classes resistant,* MARI* multiple antibiotic resistance index,* MDR* multidrug-resistant,* OP* outpatient,* IP* inpatient.

### Factors associated with gastrointestinal carriage of the CPE

#### Patient characteristics associated with CRE among children under five years

Those who presented with chills symptoms were four times more likely to have CRE compared to those without chills, cOR = 4.38, 95% CI: 1.35–14.25, *p* = 0.027. Multivariable model established that those with chills symptom were four times likely to have CRE, aOR = 4.03, 95% CI: 1.22–13.37, *p* = 0.023 as shown in Table 4.

**Table 4:** Patient characteristics associated with CRE among children under five years.

Characteristics	CRE		Total *n*(%)	cOR (95% CI)	*P*-value	aOR (95% CI)	*P*- value
	Yes *n*(%)	No (%)					
Gender							
Male	24 (58.5)	255 (51.2)	279 (51.8)	1.35 (0.71–2.57)	0.418		
Female	18 (41.5)	243 (48.8)	261 (48.2)	Ref			
Age							
≤ 24 months	27 (65.9)	378 (75.8)	405 (75.0)	0.62 (0.31–1.22)	0.188	0.66 (0.33–1.31)	0.234
25–59 months	14 (34.1)	121 (24.2)	135 (25.0)	Ref		Ref	
Symptoms							
Vomiting							
Yes	15 (36.6)	133 (26.7)	148 (27.4)	1.59 (0.82–3.09)	0.202		
No	26 (63.4)	366 (73.3)	392 (72.6)	Ref			
Chills							
Yes	4 (9.8)	12 (2.4)	16 (3.0)	4.38 (1.35–14.25)	0.027*	4.03 (1.22–13.37)	0.023*
No	38 (90.2)	486 (97.6)	524 (97.0)	Ref		Ref	
Diarrhoea							
Yes	10 (24.4)	129 (25.9)	139 (25.8)	0.92 (0.44–1.94)	0.499		
No	31 (75.6)	369 (74.1)	400 (74.2)	Ref			
Headache							
Yes	0	5 (1.0)	5 (0.9)				
No	41 (100)	492 (99.0)	533 (99.1)				
Respiratory illness							
Yes	22 (53.7)	198 (39.8)	220 (40.9)	1.75 (0.92–3.32)	0.099	1.75 (0.92–3.33)	0.090
No	19 (46.3)	299 (60.2)	318 (59.1)	Ref		Ref	
Fever							
Yes	18 (43.9)	186 (37.5)	204 (38.0)	1.30 (0.66–2.48)	0.409		
No	23 (56.1)	310 (62.5)	336 (62.0)	Ref			
Medication in last 3 months							
Yes	28 (68.3)	310 (62.1)	338 (62.6)	1.31 (0.66–2.60)	0.504		
No	13 (31.7)	189 (37.9)	202 (37.4)	Ref			
Source of medication							
Chemist	7 (25.0)	65 (21.0)	72 (21.3)	1.26 (0.51–3.08)	0.631		
Healthcare facility	21 (75.0)	245 (79.0)	266 (78.7)	Ref			
Drugs from chemist with prescription							
Yes	0	33 (50.8)	33 (45.8)				
No	7 (100)	32 (49.2)	39 (54.2)				
Gives antibiotics only when prescribed							
Yes	14 (34.1)	181 (36.4)	195 (36.2)	0.91 (0.46–1.77)	0.866		
No	27 (65.9)	316 (63.6)	343 (63.8)	Ref			
Completed last antibiotic treatment							
Yes	21 (52.5)	281 (56.4)	302 (56.1)	0.85 (0.45–1.63)	0.624		
No	21 (47.5)	217 (43.6)	238 (43.9)	Ref			
Source of drinking water							
Municipal							
Yes	39 (95.1)	456 (91.4)	495 (91.7)	1.84 (0.43–7.88)	0.563		
No	2 (4.9)	43 (8.6)	45 (8.3)	Ref			
Borehole							
Yes	2 (4.9)	43 (8.6)	45 (8.3)	0.54 (0.13–2.33)	0.563		
No	39 (95.1)	456 (91.4)	495 (91.7)	Ref			

*cOR* crude odds ratio,* CI* confidence interval,* Ref* reference category,* aOR* adjusted odds ratio.

*Significant at 0.05.

## Discussion

In this study, all carbapenem-resistant Enterobacterales (CRE) were carbapenemase producers. Similar findings were reported in a Nairobi-City County hospital in Kenya^13^. In other studies, Li and colleagues (2024) found that all CRE among adults from four provinces of China encoding carbapenemase genes^23^, while Davari et al. found 95.2% of CRE isolates from patients admitted to intensive care unit (ICU) in Southern Iran were CPE^24^. The gastrointestinal (GI) carriage rate of carbapenemase-producing Enterobacterales *(*CPE) in the current study was higher among inpatients (9.6%), than outpatients (5.9%,) under the age of five years. Transmission of CRE is frequent in healthcare settings compared to community settings^25^, with healthcare providers playing a role in the acquisition among hospitalized patients^26,27^. The carriage rate among the inpatients (9.6%) in the current study was higher than the 2.25% asymptomatic carriage reported among children (≤ 5 y) admitted in Mama Lucy Kibaki in Nairobi, Kenya^13^and 7.3% adult patients in Saint Paul’s Hospital Millennium Medical College, Addis Ababa, Ethiopia^28^. However, in our study CPE gastrointestinal carriage of 9.6% was lower than 28% documented among admitted patients in a tertiary care hospital in Egypt^29^.

Among outpatients, CPE gastrointestinal carriage was 5.9% which was lower than 9% reported among children and adult’s household contacts of CPE cases in Ontario, Canada^30^, but higher than 0.6% CRE reported carriage rate in rural communities in Vietnam^31^ and 3.6% in outpatient children in Shanghai^32^. The discordance in these study findings could result from differences in methodology and geographical variation in the epidemiology of CRE^25,33^ based on local antibiotic prescribing practices, the misuse or overuse of carbapenems, personal and environmental hygiene, and insufficient infection prevention and control (IPC) strategies within healthcare settings. The carbapenem resistance is mediated by carbapenemases, a group of enzymes encoded on mobile genetic elements, allowing rapid spread and extensive distribution of the resistance traits among bacterial pathogens, escalating antimicrobial resistance (AMR)^34^. In the current study setting, the high gastrointestinal of CPE carriage represents a significant public health threat due to the potential for heightened transmission, healthcare-associated outbreaks, and community infections with limited treatment options. Our study finding underscores the urgent need for a multifaceted approach involving improved infection control practices, antimicrobial stewardship, surveillance systems, and international collaboration in line with global and international action plans on antimicrobial resistance.

In the current research, the CPE spectrum included five bacterial species recovered from outpatients and four from inpatients. Carbapemase-producing (CP) *Enterobacter cloacea* and CP *Klebsiella variicola* were not isolated from outpatients, while CP *Citrobacter freundii*, CP *Enterobacter bugandensis,* and CP *Proteus mirabilis* were missing from the inpatient children. The bacterial spectrum in our study differs from that obtained from randomly selected infants (< 1 year), children (1–17 years), and adults (≥ 18 years) from a hospital and associated communities in Guatemala during the coronavirus disease 2019 (COVID-19) pandemic^35^. We also found a co-carriage of *E. coli* and *Klebsiella pneumoniae* among inpatients (0.4%), consistent with a study that reported gastrointestinal carriage with multiple CRE isolates in the same patients^36^.

*Escherichia coli* was the predominant CPE in both inpatients (84.6%) and outpatients ( 62.5%). This finding is consistent with those of studies in the Northwest, Ethiopia^37^, India^38^, and Guatemala^35^, where *E. coli* was the most common gastrointestinal carbapenemase-resistant isolate. Contrarily to our study finding, *Enterobacter cloacae* was the predominant CPE isolate reported in Addis Ababa, Ethiopia^28^, and *E. coli* and *Enterobacter cloacae subsp dissolvens* in a recent study in Nairobi, Kenya^13^. The spectrum of carbapenemase-resistant bacteria and level of resistance can vary geographically depending on the genetic makeup of bacterial populations, differences in antibiotic usage patterns, adherence to infection control in healthcare settings and antimicrobial stewardship policies, human travel and migration, and environmental factors such as sanitation practices and agricultural antibiotic use. Effective interventions to combat the spread of the carbapenemase-producing bacteria found in this study remain critical.

In the current study, CPEC resistance to third-generation cephalosporins (3GCs), including ceftriaxone, ceftazidime and cefotaxime, was 100% in both outpatient and inpatient department. Resistance to aminoglycosides (gentamicin (GEN) and amikacin (AMK)) was highest in CPEC isolates from inpatients (32%) compared with those from outpatients (10%). All the isolates from inpatient (100%) and 80% of those recovered from outpatient were multidrug-resistant. Previous studies have documented higher drug-resistant bacteria in hospitals than in community settings^39,40^. Hospitalized patients are more likely to harbour drug-resistant bacteria due to frequent antibiotic use, a high number of vulnerable patient populations, and the complex and dynamic environment that creates a conducive niche for the emergence and spread of drug-resistant organisms.

Our study finding on 3GC resistance was similar to that reported in CRE stool isolates from adults in China^23^, children in Shanghai^41^ and infants, children and adults in Guatemala^35^. Other studies have reported lower resistance levels of ceftriaxone (0%) in Hunan, China^42^ and ceftriaxone (59.6%), cefotaxime (67.3%), and ceftazidime (94.2%) in Northwest Ethiopia^37^ among CRE stool isolates from hospitalized patients. Extended-spectrum beta-lactamase (ESBL)-producing Enterobacterales, defined by resistance to 3GCs, fourth-generation cephalosporins (4GCs), and monobactams, are among the World Health Organization (WHO) top-priority pathogens that require urgent development of antibiotics^43^. In addition to 3GC resistance, CPEC isolates in the current study were 60–91% cefepime (4GCs)-resistant and 100% monobactam (aztreonan)-resistant, suggesting the presence of ESBL-producing phenotypes.

The 3GC resistance in the current study represents a significant public health threat since these isolates were multidrug-resistant and produced carbapenemases, enzymes that inactivate the carbapenems, the antibiotics considered ‘the last resort’ for treating multidrug-resistant organisms. Multiple antibiotic resistance indices (MARI) of the isolates were high, ranging from 0.23 to 0.46. The high MARI (> 0.2) in the current study indicated that the CPEC isolates had prior exposure to multiple antibiotic environments and were more likely to cause difficult-to-treat infections^44^. However, these isolates remained susceptible to colistin, a finding consistent with Liu et al. in Hunan, China^42^. Elsewhere, colistin resistance in CRE stool isolates is documented among adults (1.47%) in China^45^ and children (9.4%) in Shanghai^41^. Our study finding highlights the clinical relevance of colistin and the need for ongoing antibiotic stewardship, infection control, and public health strategies to combat AMR resistance and secure public health in current and similar settings.

Children (≤ 5 years) presenting with chills symptoms were four times more likely to have CPE gastrointestinal colonization when compared with those without such a symptom. Chills are traditionally associated with severe bacterial infections and bacteremia^46^. Intestinal CRE colonization may precede infection among susceptible individuals, such as those with weakened immunity, admitted to intensive care units (ICU), artificially ventilated, and catheterization^17^. Additionally, gastrointestinal bacteria that have acquired antimicrobial resistance genes can degrade antibiotics, shielding pathogens from the bactericidal effects of antibiotics^47^. Enhanced environmental cleaning, strict adherence to hand hygiene protocols, patient isolation, and screening for colonization are required to prevent CRE transmission during hospital stays.

This study had some limitations in that we did not elucidate the molecular characteristics of the isolates due to financial constraints and identify the factors associated with the colonization among the outpatients, perhaps due to a small study sample.

## Conclusion

We observed a significant gastrointestinal carriage of carbapenem-resistant Enterobacterales (CRE) that were carbapenemase producers among inpatient and outpatient children (≤ 5 years), with chills as a predictor of colonization among the hospitalized participants. It is critical to conduct antimicrobial resistance surveillance, through routine screening of clinical isolates for CPE production and adhere to antimicrobial stewardship policies to control CRE dissemination in the current study setting and beyond.

## Electronic supplementary material

Below is the link to the electronic supplementary material.

Supplementary Material 1

## Data Availability

The datasets utilized in this study can be acquired from the corresponding author upon request.
