# Supplementary material for: Gastrointestinal carriage of carbapenemase-producing enterobacterales among inpatient and outpatient children in Kenya
Source: Sci Rep. 2024 Dec 28;14:30684. doi: 10.1038/s41598-024-78059-1 (PMC11680766; doi:10.1038/s41598-024-78059-1)
Supplement: Supplementary file 1 — Supplementary Material 1 [file 41598_2024_78059_MOESM1_ESM.docx]

**Supplementary Table S1:** *Factors associated with the gastrointestinal carriage of the CPE among children (≤5 years) among the inpatients*

| Variables | Total n(%) | CPE  Present, n(%) | CPE  Absent, n(%) | cOR(95%CI) | P-value | aOR(95% CI) | P-value |
| --- | --- | --- | --- | --- | --- | --- | --- |
| Gender | | | | | | | |
| Male | 144(53.3) | 16(61.5) | 127(52.3) | 1.46(0.64 - 3.35) | 0.413 |  |  |
| Female | 126(46.7) | 10(38.5) | 116(47.7) | Ref |  |  |  |
| Age | | | | | | | |
| ≤24 months | 198(73.3) | 16(61.5) | 182(74.6) | 0.55(0.24 - 1.26) | 0.165 | 0.59(0.19 - 1.78) | 0.347 |
| 25 - 59 months | 72(26.7) | 10(38.5) | 62(25.4) | Ref |  | Ref |  |
| Clinical presentations | | | | | | | |
| Vomiting |  |  |  |  |  |  |  |
| *Yes* | 99(36.7) | 10(38.5) | 89(36.5) | 1.09(0.47 - 2.50) | 0.834 |  |  |
| *No* | 171(63.3) | 16(61.5) | 155(63.5) | Ref |  |  |  |
| Chills |  |  |  |  |  |  |  |
| Yes | 13(4.8) | 3(11.5) | 10(4.1) | 3.05(0.78 - 11.89) | 0.118 | 10.57(1.84 - 60.75) | 0.008* |
| No | 257(95.2) | 23(88.5) | 234(95.9) | Ref |  | Ref |  |
| Diarrhoea |  |  |  |  |  |  |  |
| Yes | 97(35.9) | 8(30.8) | 89(36.5) | 0.77(0.32 - 1.85) | 0.67 |  |  |
| No | 173(64.1) | 18(69.2) | 155(63.5) | Ref |  |  |  |
| Headache |  |  |  |  |  |  |  |
| Yes | 2(0.7) | 0 | 2(0.8) |  |  |  |  |
| No | 267(99.3) | 26(100) | 241(99.2) |  |  |  |  |
| RT illness |  |  |  |  |  |  |  |
| *Yes* | 144(53.3) | 17(65.4) | 127(52.0) | 1.74(0.75 - 4.06) | 0.22 |  |  |
| *No* | 126(46.7) | 9(34.6) | 117(48.0) | Ref |  |  |  |
| Fever |  |  |  |  |  |  |  |
| *Yes* | 136(50.6) | 14(53.8) | 122(50.2) | 1.16(0.51 - 2.60) | 0.837 |  |  |
| *No* | 133(49.4) | 12(46.2) | 121(49.8) | Ref |  |  |  |
| History of antibiotics use in last 3 months | | | | | | | |
| Yes | 192(71.1) | 18(69.2) | 174(71.3) | 0.91(0.38 - 2.18) | 0.822 |  |  |
| No | 78(28.9) | 8(30.8) | 70(28.7) | Ref |  |  |  |
| Source of antibiotics | | | | | | | |
| Chemist | 35(18.2) | 6(33.3) | 29(16.7) | 2.50(0.87 - 7.20) | 0.105 | 1.98(0.62 - 6.34) | 0.249 |
| Hospital | 157(81.8) | 12(66.7) | 145(83.3) | Ref |  |  |  |
| Use clinician-prescribed antibiotics | | | | | | | |
| Yes | 112(41.8) | 10(38.5) | 102(42.1) | 0.86(0.37 - 1.97) | 0.835 |  |  |
| No | 156(58.2) | 16(61.5) | 140(57.9) | Ref |  |  |  |
| Completed last antibiotic dose | | | | | | | |
| Yes | 106(39.6) | 10(40.0) | 96(39.5) | 1.02(0.44 - 2.37) | 0.561 |  |  |
| No | 162(60.4) | 15(60.0) | 147(60.5) | Ref |  |  |  |
| Sources of domestic water | | | | | | | |
| Municipal water |  |  |  |  |  |  |  |
| *Yes* | 241(89.3) | 24(92.3) | 217(88.9) | 1.49(0.33 - 6.67) | 0.452 |  |  |
| *No* | 29(10.7) | 2(7.7) | 27(11.1) | Ref |  |  |  |
| Borehole |  |  |  |  |  |  |  |
| *Yes* | 29(10.7) | 2(7.7) | 27(11.1) | 0.67(0.15 - 2.99) | 0.452 |  |  |
| *No* | 241(89.3) | 24(92.3) | 217(88.9) | Ref |  |  |  |

**CPE:** carbapenemase-producing *Entrobacteriaeceae***, cOR:** Crude Odds ratio, **CI:** Confidence interval, **Ref:** Reference category, **aOR:** adjusted odds ratio, **RT** respiratory tract, ***** Significant association at 0.05.

**Supplementary Table S2:** *Factors associated with the gastrointestinal carriage of the CPE among children (≤5 years) among the outpatients*

| Characteristics | Total n(%) | CPE | | cOR(95%CI) | P-value |
| --- | --- | --- | --- | --- | --- |
|  |  | **Yes n(%)** | **No n(%)** |  |  |
| Gender |  |  |  |  |  |
| Male | 136(50.4) | 8(53.3) | 128(50.2) | 1.13(0.40 - 3.22) | 0.512 |
| Female | 134(49.6) | 7(46.7) | 127(49.8) | Ref |  |
| Age |  |  |  |  |  |
| ≤24 months | 207(76.7) | 11(73.3) | 196(76.9) | 0.83(0.25 - 2.70) | 0.756 |
| 25 - 59 months | 63(23.3) | 4(26.7) | 59(23.1) | Ref |  |
| Clinical presentation | | | | | |
| Vomiting |  |  |  |  |  |
| *Yes* | 49(18.1) | 5(33.3) | 44(17.3) | 2.40(0.78 - 7.36) | 0.259 |
| *No* | 221(81.9) | 10(66.7) | 211(82.7) | Ref |  |
| Chills |  |  |  |  |  |
| *Yes* | 3(1.1) | 1(6.7) | 2(0.8) | 9.0(0.77 - 105.35) | 0.259 |
| *No* | 266(98.9) | 14(93.3) | 252(99.2) | Ref |  |
| Diarrhoea |  |  |  |  |  |
| *Yes* | 42(15.6) | 2(13.3) | 40(15.7) | 0.82(0.18 - 3.79) | 0.756 |
| *No* | 227(84.4) | 13(86.7) | 214(84.3) | Ref |  |
| Headache |  |  |  |  |  |
| *Yes* | 3(1.1) | 0 | 3(1.2) |  |  |
| *No* | 266(98.9) | 15(100) | 251(98.8) |  |  |
| Respiratory |  |  |  |  |  |
| *Yes* | 76(28.4) | 5(33.3) | 71(28.1) | 1.28(0.42 - 3.88) | 0.769 |
| *No* | 192(71.6) | 10(66.7) | 182(71.9) | Ref |  |
| Fever |  |  |  |  |  |
| *Yes* | 68(25.4) | 4(26.7) | 64(25.3) | 1.07(0.33 - 3.49) | 0.556 |
| *No* | 200(74.6) | 11(73.3) | 189(74.7) | Ref |  |
| History of antibiotics use in the last 3 months | | | | | |
| Yes | 146(54.1) | 10(66.7) | 136(53.3) | 1.75(0.58 - 5.36) | 0.426 |
| No | 124(45.9) | 5(33.3) | 119(46.7) | Ref |  |
| Source of antibiotics | | | | | |
| Chemist | 37(25.3) | 1(10.0) | 36(26.5) | 0.31(0.04 - 2.52) | 0.452 |
| Healthcare facility | 109(74.7) | 9(90.0) | 100(73.5) | Ref |  |
| Use clinician-prescribed antibiotics | | | | | |
| Yes | 83(30.7) | 4(26.7) | 79(31.0) | 0.81(0.25 - 2.62) | 0.488 |
| No | 187(69.3) | 11(73.3) | 176(69.0) | Ref |  |
| Completed the last antibiotic dose | | | | | |
| Yes | 196(72.6) | 11(73.3) | 185(72.5) | 1.04(0.32 - 3.38) | 0.606 |
| No | 74(27.4) | 4(26.7) | 70(27.5) | Ref |  |
| Sources of domestic water | | | | | |
| Municipal water |  |  |  |  |  |
| *Yes* | 254(94.1) | 15(100) | 239(93.7) |  |  |
| *No* | 16(5.9) | 0 | 16(6.3) |  |  |
| Borehole water |  |  |  |  |  |
| *Yes* | 16(5.9) | 0 | 16(6.3) |  |  |
| *No* | 254(94.1) | 15(100) | 239(93.7) |  |  |

**CPE:** carbapenemase-producing *Entrobacteriaeceae***, cOR:** Crude Odds ratio, **CI:** Confidence interval, **Ref:** Reference category, **aOR:** adjusted odds ratio, **RT** respiratory tract, *****Significant association at 0.05.
